# Lessons Learned from Programmatic Gains in HIV Service Delivery During the COVID-19 Pandemic — 41 PEPFAR-Supported Countries, 2020

**DOI:** 10.15585/mmwr.mm7112a2

**Published:** 2022-03-25

**Authors:** Kiva A. Fisher, Sadhna V. Patel, Neha Mehta, Andrea Stewart, Amy Medley, Emily Kainne Dokubo, Judith D. Shang, Janell Wright, Jose Rodas, Shirish Balachandra, Francois Kitenge, Minlangu Mpingulu, Macarena C. García, Luis Bonilla, Silas Quaye, Michael Melchior, Ketmala Banchongphanith, Kunjanakorn Phokhasawad, Kondwani Nkanaunena, Alice Maida, Aleny Couto, Jose Mizela, Jahun Ibrahim, Ogbanufe Obinna Charles, Samuel S. Malamba, Canisious Musoni, Alex Bolo, Sudhir Bunga, Rangsima Lolekha, Wiphawee Kiatchanon, Ramona Bhatia, Chi Nguyen, John Aberle-Grasse, PEPFAR Strategic Information Study Group, PEPFAR Strategic Information Study Group, Quoc Nguyen, Phuong N. Nguyen, Dan Williams, Michael DeKlerk, Tuli Nakanyala, Nicasky Celestin, Ngwarai Sithole, Trust Chiguvare, Chiedza Marisa, Kelsey Mirkovic, Evelyn Muthama, Davies Kimanga, Abraham Katana, Apolonia Aoko, Andrew Auld, Masford Banda, Laurence Gunde, Muluken Kaba, Dumbani Kayira, Elizabeth Kampira, Evelyn Kim, Mischeck Luhanga, Gillian Nkhalamba, Mtemwa Nyangulu, Nellie Wadonda-Kabondo, Rose Nyirenda, Andreas Jahn, Suchunya Aungkulanon, Benjamas Baipluthong, Lindsay Templin, Joseph Lara, Michelle Li, Samuel Kudhlande, Kelly-Ann Gordon-Johnson, Sasha Martin, Mduduzi Ndlovu, Colince Leonard Keleko, Elizabeth Manuela Kamga, Esther Lyonga, Ebako Ndip Takem, Eva Matiko, Kokuhumbya J. Kazaura, Coline Mahende

**Affiliations:** ^1^Division of Global HIV & TB, Center for Global Health, CDC; ^2^CDC, Cameroon; ^3^CDC, Central America; ^4^CDC, Côte d’Ivoire; ^5^CDC, Democratic Republic of the Congo; ^6^CDC, Dominican Republic; ^7^CDC, Ghana; ^8^Ministry of Public Health, Laos; ^9^CDC, Laos; ^10^CDC, Malawi; ^11^Mozambique Ministry of Health; ^12^CDC, Mozambique; ^13^Global Fund/NACA Resilient and Sustainable Systems for Health; ^14^CDC, Nigeria; ^15^CDC, Rwanda; ^16^CDC, South Sudan; ^17^CDC, Thailand; ^18^CDC, Vietnam.; CDC-Vietnam; CDC-Vietnam; CDC-Namibia; CDC-Namibia; CDC-Namibia; CDC-Haiti; Ministry of Health and Child Care, Zimbabwe; CDC-Zimbabwe; CDC-Zimbabwe; CDC-Zimbabwe; DGHT, CDC-Kenya; DGHT, CDC-Kenya; CDC-Kenya; DGHT, CDC-Kenya; CDC-Malawi; CDC-Malawi; CDC-Malawi; CDC-Malawi; CDC-Malawi; CDC-Malawi; CDC-Malawi; CDC-Malawi; CDC-Malawi; CDC-Malawi; CDC-Malawi; Department of HIV & AIDS, Malawi Ministry of Health; ITECH-Malawi; CDC Thailand/Lao PDR; CDC Thailand/Lao PDR Programs; CDC-Mozambique; USAID-Mozambique; CDC-Eswatini; CDC-Eswatini; CDC-Caribbean Regional Office; PEPFAR Coordinating Office, Caribbean Regional Program; CDC-South Africa; CDC Cameroon; CDC-Cameroon; CDC-Cameroon; CDC-Cameroon; CDC-Tanzania; CDC-Tanzania; CDC-Tanzania

The U.S. President’s Emergency Plan for AIDS Relief (PEPFAR) supports country programs in identifying persons living with HIV infection (PLHIV), providing life-saving treatment, and reducing the spread of HIV in countries around the world (*1*,*2*). CDC used Monitoring, Evaluation, and Reporting (MER) data* to assess the extent to which COVID-19 mitigation strategies affected HIV service delivery across the HIV care continuum^†^ globally during the first year of the COVID-19 pandemic. Indicators included the number of reported HIV-positive test results, the number of PLHIV who were receiving antiretroviral therapy (ART), and the rates of HIV viral load suppression. Percent change in performance was assessed between countries during the first 3 months of 2020, before COVID-19 mitigation efforts began (January–March 2020), and the last 3 months of the calendar year (October–December 2020). Data were reviewed for all 41 countries to assess total and country-level percent change for each indicator. Then, qualitative data were reviewed among countries in the upper quartile to assess specific strategies that contributed to programmatic gains. Overall, positive percent change was observed in PEPFAR-supported countries in HIV treatment (5%) and viral load suppression (2%) during 2020. Countries reporting the highest gains across the HIV care continuum during 2020 attributed successes to reducing or streamlining facility attendance through strategies such as enhancing index testing (offering of testing to the biologic children and partners of PLHIV)^§^ and community- and home-based testing; treatment delivery approaches; and improvements in data use through monitoring activities, systems, and data quality checks. Countries that reported program improvements during the first year of the COVID-19 pandemic offer important information about how lifesaving HIV treatment might be provided during a global public health crisis.

During 2020, 41 countries received PEPFAR support for direct HIV service delivery.^¶^ To determine gains in HIV service delivery, MER indicators were analyzed to identify programmatic changes in 1) the number of reported positive HIV test results, 2) the number of PLHIV receiving ART, and 3) the percentage of PLHIV receiving ART with suppressed HIV viral load** during 2020 to assess change before and during the first year of the COVID-19 pandemic. The number of sites ranged from three to 1,520 per country. The analysis was limited to sites within each country that reported indicator data during both periods. The number of treatment sites in each country that reported during both periods was proportional to the size of the PEPFAR ART program. Overall percent change for all 41 countries from January–March to October–December 2020^††^ was calculated for each of the three indicators. The percent change for each indicator was further analyzed for countries in the highest quartile for each indicator. A thematic analysis was conducted using qualitative MER narratives for each indicator among countries in the upper quartile to identify specific adaptive strategies that were reported to contribute to gains for each indicator. This activity was reviewed by CDC and was conducted consistent with applicable federal law and CDC policy.^§§^

Among all 41 countries, programmatic gains (i.e., positive percent changes) were observed in the number of PLHIV reported to be currently receiving treatment and the percentage who had achieved viral load suppression before and during the COVID-19 pandemic. Positive percent change among all sites reported across the 41 countries was reported for the number of PLHIV currently receiving treatment (5%) and for HIV viral load suppression (2%). However, an overall negative percent change (−19%) was reported in HIV-positive test results (Figure). Percent change at the country level varied by indicator. Positive percent change was observed in the number of HIV-positive test results reported in 16 (39%) countries, the number of persons receiving ART in 36 (88%) countries, and HIV viral load suppression rates in and 29 of 34 countries with reported data^¶¶^ (85%).

**FIGURE:**
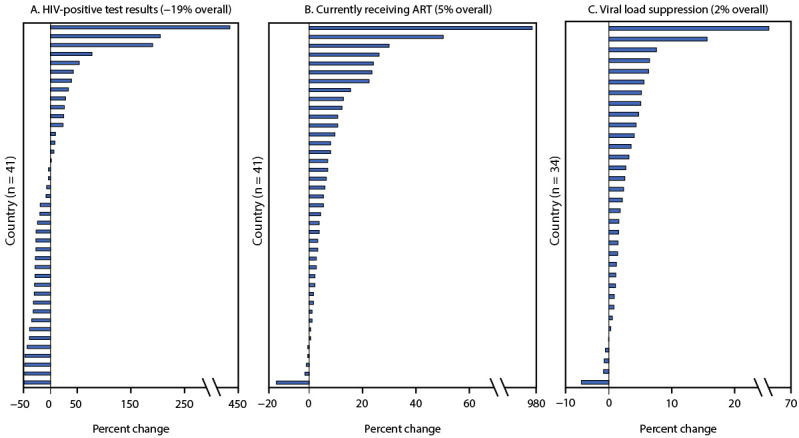
Percent change in HIV-positive test results* (A), number of persons with HIV receiving antiretroviral therapy^†^ (B), and rates of viral load suppression^§^ (C) — U.S. President’s Emergency Plan for AIDS Relief, 41 countries, January–March to October–December 2020 **Abbreviations**: ART = antiretroviral therapy; UNAIDS = Joint United Nations Programme on HIV/AIDS; WHO = World Health Organization. * Number of persons who received a positive HIV test result. https://datim.zendesk.com/hc/en-us/articles/360000084446-MER-Indicator-Reference-Guides ^†^ Number of adults and children who are currently receiving ART in accordance with the nationally approved treatment protocol (or WHO/UNAIDS standards) at the end of the reporting period. https://datim.zendesk.com/hc/en-us/articles/360000084446-MER-Indicator-Reference-Guides ^§^ Percentage of viral load suppression. Only sites that are in the highest quartile in both prepandemic (January–March 2020) and pandemic (October–December 2020) periods are included. Thirty-four of the 41 countries were included; seven countries did not report data for the viral load indicator.

Twenty-three (56%) countries were in the upper quartile for at least one indicator, and six (15%) reported the highest overall percent change for two of the three indicators. One country (Nicaragua) showed gains across all three indicators. Countries reporting increases for any of the three indicators spanned all regions where PEPFAR supports HIV programs, including eight countries in West Africa, three in East Africa, two in Southern Africa, five in Central America, four in Asia, and one in the Caribbean. Among countries in the highest quartile, a median increase of 43% was reported in the number of HIV-positive test results identified (range = 25%–430%), 23% in the number of PLHIV receiving ART (range = 11%–965%), and 6% in HIV viral load suppression rates (range = 5%–62%) from January–March to October–December 2020 (during the COVID-19 pandemic) (Table).

**TABLE:** Countries with upper quartile gains in HIV service delivery* — U.S. President’s Emergency Plan for AIDS Relief, 23 countries, January–March and October–December 2020

Country	No. of sites	Jan–Mar 2020, no. or %	Oct–Dec 2020, no. or %	% Change
**No. of HIV-positive test results^†^**				
Indonesia	37	132	700	430.3
Laos	3	63	192	204.8
Liberia	11	180	523	190.6
El Salvador	10	102	181	77.5
Panama	7	65	100	53.8
Nicaragua	5	47	67	42.6
Vietnam	69	1,987	2,767	39.3
Nigeria	1,288	77,099	102,742	33.3
Dominican Republic	17	1,702	2,181	28.1
Rwanda	146	1,450	1,825	25.9
Burkina Faso	17	1,022	1,276	24.9
**No. of PLHIV currently receiving ART^§^**				
Liberia	12	1,023	10,895	965.0
Nicaragua	6	886	1,328	49.9
Nigeria	1,435	907,653	1,177,770	29.8
Ghana	44	12,181	15,353	26.0
Democratic Republic of the Congo	516	134,107	166,081	23.8
Thailand	36	45,159	55,770	23.5
Togo	24	28,433	34,777	22.3
South Sudan	65	27,926	32,267	15.5
Mozambique	1,520	1,224,808	1,378,579	12.6
Laos	7	6,699	7,517	12.2
Senegal	4	3,347	3,708	10.8
**% Viral load suppression^¶^**				
Nicaragua	5	51	83	62.1
Cameroon	142	78	90	15.6
Mozambique	563	82	88	7.6
Panama	8	73	77	6.5
Guatemala	8	83	88	6.3
Côte d'Ivoire	490	84	88	5.6
Democratic Republic of the Congo	510	88	93	5.2
Honduras	6	85	90	5.1
Malawi	641	89	93	4.7

**Abbreviations:** ART  **=** antiretroviral therapy; PLHIV = persons living with HIV; UNAIDS = Joint United Nations Programme on HIV/AIDS; WHO = World Health Organization.

* Percent change in number of HIV-positive test results, persons with HIV on antiretroviral treatment, and percentage with viral load suppression.

^†^ Number of persons who received a positive HIV test result. https://datim.zendesk.com/hc/en-us/articles/360000084446-MER-Indicator-Reference-Guides

^§^ Number of adults and children who are currently receiving ART in accordance with the nationally approved treatment protocol (or WHO/UNAIDS standards) at the end of the reporting period. https://datim.zendesk.com/hc/en-us/articles/360000084446-MER-Indicator-Reference-Guides

^¶^ Percentage of viral load suppression. Only sites in the highest quartile in both prepandemic (January–March 2020) and pandemic (October–December 2020) periods are included. Thirty-four of the 41 countries were included; seven countries did not report data for the viral load indicator.

Review of quarterly aggregate MER narrative reports among countries in the upper quartile of positive HIV tests reported and case finding approaches attributed success to enhancing index testing and community- and home-based testing approaches tailored to geographic locations and populations at increased risk for HIV. Country programs in the upper quartile for increases in HIV treatment during the analytic time frame were associated with policy shifts toward multimonth dispensing of HIV treatment; streamlining facility visits (e.g., appointment spacing); facilitating community, peer, and home ART delivery options; improvements in data systems and management processes (e.g., patient tracking and tracing and site-level monitoring); and the use of telecommunication methods for improving client services. Gains in HIV viral load suppression among upper quartile programs were attributed to addressing a backlog of sample testing associated with the shifting priorities early in COVID-19 responses and reagent stockouts as well as improved patient monitoring and data quality. Tracking and tracing efforts among PLHIV with elevated viremia and aligning viral load testing with medication pick-up were reported as strategies to improve rates of viral load suppression.

## Discussion

During 2020, many countries experienced disruptions to routine health care service delivery and challenges with infrastructure, human resources, and medical supplies as a result of the COVID-19 pandemic. Early estimates projected negative pandemic-related impacts on HIV service delivery (*3*). This report highlights the capacity of PEPFAR-supported countries to adapt HIV programs to the COVID-19 pandemic, particularly related to gains in HIV treatment and viral load suppression. Strategies reported among countries with gains in HIV programming included finding effective ways to reduce the frequency and duration of facility visits; streamlining service provision through community approaches, telecommunications, or messaging services; and enhancing quality and use of MER data and improving data systems for program improvement. PLHIV who are receiving ART experience less severe outcomes related to COVID-19 infection than do those who are not receiving ART (*4*,*5*), making access to HIV testing and treatment services critically important as the COVID-19 pandemic continues.

Countries reporting increases in identifying new HIV infections attributed accomplishments to increasing community- and home-based testing and index testing approaches. Case finding efforts based on community-based and index testing have historically provided opportunities for early identification of PLHIV and reaching persons outside of facility settings (*6*,*7*). Scaling up community-based testing during the COVID-19 pandemic might have helped relieve some of the strain on health care infrastructure by reducing the overall number of persons visiting facilities for HIV testing services. Gains in identification of new HIV-positive persons was reported by the lowest percentage (39%) of countries, compared with gains in HIV treatment (88%) and viral load suppression (85%), in line with PEPFAR recommendations*** to focus efforts on retaining known PLHIV on treatment.

HIV treatment gains were reported from a variety of programmatic strategies, including facility-, community-, peer-, and telecommunications-based approaches; improved data use related to patient tracking and tracing activities; and site-level monitoring. Facilities reported activities that reduced onsite attendance such as community home delivery models. Multimonth dispensing of ART, a strategy known to be successful in providing ART to PLHIV among countries affected by COVID-19 (*8*), was also used by PEPFAR programs. Reports of viral load testing coverage being negatively affected across PEPFAR-supported countries early in the COVID-19 pandemic have been described (*9*). Countries were able to align laboratory services with current SARS-CoV-2 testing needs to provide increases in HIV viral load testing activities over time (*9*); several countries were able to regain losses in viral load testing coverage, as well as show gains in viral load suppression, by focusing on tracking and tracing efforts among PLHIV with high levels of viremia, addressing stockout and testing backlog challenges, and aligning viral load testing with medication pickup.

The findings in this report are subject to at least five limitations. First, data are cross-sectional and reported quarterly in aggregate, precluding the ability to monitor and track persons across time. Second, data quality varies across country programs, and narrative reports were not systematically collected. Third, timing of the effect of COVID-19 on SARS-CoV-2 testing capacity and implemented mitigation measures has varied at the country level. The time frame selected for this analysis covers all reporting quarters within the calendar year to account for COVID-19 pandemic fluctuations and precedes any possible effects related to national COVID-19 vaccination rollout. Fourth, country context can affect overall results based on the capacity to adapt local infrastructures and to make programmatic shifts, including program approaches and strategies, or changes in implementing partners. To reduce the potential effects of programmatic shifts, only PEPFAR-supported facilities that reported data during both periods were included in the analysis. Finally, some countries are reaching HIV epidemic control and therefore might not have had extensive programmatic improvements, and the maintenance of those gains would not have been reflected in positive percent change in indicators. Given that lower percent change did not inherently represent reductions in program performance, the narrative analysis was restricted to countries in the upper quartile of percent change during the specified time frame. The number of sites ranged widely among countries; however, the numbers are proportional to the country size and PEPFAR ART program. Although this variation might have affected the magnitude of overall percent change across indicators, particularly in the smallest programs, the direction of change provided valuable information in assessing programmatic gains across the HIV care continuum.

Qualitative data were reviewed from countries that reported the highest percentage of programmatic gains to help identify specific strategies that might have improved HIV service delivery, considering the stress placed on HIV programs as countries worked toward mitigating SARS-CoV-2 transmission. Programs can learn from these strategies and assess their implementation feasibility to help develop sustainable activities as well as adapt programs during a global health crisis. These findings demonstrate how community- and home-based approaches, in conjunction with improving data use for program improvement, can effectively reduce facility visits in PEPFAR-supported countries and therefore help mitigate SARS-CoV-2 transmission while preserving life-saving services for PLHIV.

**Summary**

**What is already known about the topic?**

The COVID-19 pandemic has affected health care infrastructure worldwide.

**What is added by this report?**

In 41 U.S. President’s Emergency Plan for AIDS Relief (PEPFAR)–supported countries, overall gains were observed in HIV treatment (5%) and viral load suppression (2%) during the first year of the COVID-19 pandemic. Among countries with the largest overall programmatic gains, strategies that facilitated HIV program improvements included enhanced index testing and community- and home-based testing; multimonth dispensing of medications; streamlining clinic visits; aligning medication pick-up with viral load testing; and improvements in data use.

**What are the implications for public health practice?**

Lessons learned from PEPFAR-supported countries reporting the most programmatic progress in HIV programs provide important insights into strategies that can be used to realize programmatic gains during a global health crisis.

## Footnotes

* https://www.state.gov/wp-content/uploads/2019/10/PEPFAR-MER-Indicator-Reference-Guide-Version-2.4-FY20.pdf

† https://www.cdc.gov/hiv/pdf/library/factsheets/cdc-hiv-care-continuum.pdf

§
https://apps.who.int/iris/bitstream/handle/10665/251655/9789241549868-eng.pdf

¶ Direct service delivery for HIV treatment support from PEPFAR is defined as both provision of critical personnel or commodities and support to improve the quality of services through site visits as often as deemed necessary by the partner and country team. Eight countries were excluded from the analysis because PEPFAR exclusively provides nondirect service delivery support (i.e., technical assistance only) for treatment services.

** MER indicators: number of persons receiving a positive HIV test result, number of persons currently receiving antiretroviral therapy, and rate of viral load suppression. The viral load indicator collects information among persons who have been receiving ART for ≥3 months and assesses suppression among those who have had a test in the past 12 months. HIV viral load suppression is defined as having a documented viral load result of <1,000 HIV RNA copies/ml within the past 12 months. Because of reporting requirements, data on viral load might include data from previous reporting quarters and fiscal years. MER narrative reports are collected during each reporting period and include qualitative information to supplement the values reported for the indicator.

†† Data analysis was conducted using data reported from facilities during January–March and October–December 2020 to provide appropriate comparison of results before and during the first year of the COVID-19 pandemic. Community sites were included for the HIV testing indicator. This period was chosen to avoid any possible impact of COVID-19 vaccination rollout and avoid substantive programmatic changes that could take place outside a single calendar year.

§§ 45 C.F.R. part 46, 21 C.F.R. part 56; 42 U.S.C. Sect. 241(d); 5 U.S.C. Sect. 552a; 44 U.S.C. Sect. 3501 et seq.

¶¶ Thirty-four of 41 countries were included; seven countries did not report data for the viral load indicator.

*** During the COVID-19 pandemic, PEPFAR technical guidance focused efforts on continuity of treatment for PLHIV. Specific testing guidance was provided to maintain protection against COVID-19 for all persons supporting programmatic efforts related to HIV testing based on local and government policies, with the acknowledgment that testing services might be affected because of COVID-19 mitigation measures.
